# Methods for restoration of ki67 antigenicity in aged paraffin tissue blocks

**DOI:** 10.1007/s00418-021-01987-w

**Published:** 2021-04-10

**Authors:** Federica Grillo, Michela Campora, Simona Pigozzi, Silvia Bonadio, Luca Valle, Jacopo Ferro, Michele Paudice, Beatrice Dose, Luca Mastracci

**Affiliations:** 1 Anatomic Pathology Unit, Department of Surgical Sciences and Integrated Diagnostics (DISC), University of Genova, IRCCS AOU San Martino IST, Largo Rosanna Benzi, 10, 16132 Genoa, Italy; 2 Anatomic Pathology, Ospedale Policlinico San Martino IRCCS, Genova, Italy

**Keywords:** Ki67, Immunohistochemistry, Antigen retrieval, Antigen decay, Tissue block storage, Archival pathology

## Abstract

Pathology archives are a treasure trove of paraffin embedded tissue spanning many years and covering a wide variety of tissues and diseases. The possibility of using old archival formalin fixed paraffin embedded (FFPE) tissues for diagnostic updates and research projects is a widespread need and it requires archives of stable, well-preserved samples. Immunohistochemistry performed on old archival paraffin blocks may give unreliable results, in particular for some antigens, such as Ki67. In consideration of this phenomenon, our aim is to comprehensively test and identify methods which may be used to obtain Ki67 immunohistochemical reactions of good quality from old archival FFPE blocks. Various methods were tested in order to evaluate their possible efficacy in increasing Ki67 immunointensity in a collection of 40-year-old, archival blocks including re-embedding, with deeper sectioning of tissue from the block and increasing heat-based pretreatment times (20 cases) and re-processing (20 cases). All reactions were performed using an automated immunostainer and Ki67 stained immunosections compared using a visual colour-based scale (the first immunostained section was considered as baseline). The combination of deep sectioning (1000 µM) and prolonged heat-based pretreatment (64 min) markedly increased immunoreactivity for Ki67. Re-embedding and reprocessing did not have a significant effect. Large tissue samples showed heterogeneity of Ki67 immunoexpression between the periphery of the sample and the central area. In conclusion, the study defines a useful protocol to increase antigen retrieval applicable to dated archival tissues.

## Introduction

Antigen masking related to tissue processing and, in particular, to formalin fixation is a well-known problem, which may have far reaching consequences on the outcome of immunohistochemical reactions and which have been discussed for many years (Puchtler et al. 1985). Over the years, different antigen retrieval methods, based on the heat or the use of low concentration enzymes, have been developed and these are effective in antigen restoration (Yamashita et al. 2017). In addition to variable preanalytic factors, problems in antigen availability may also be associated with other issues in the postprocessing phase.

In particular, ageing of unstained sections has been shown to be an important factor which may render antigen retrieval less effective (Grillo et al. 2015). Presectioned tissue on slides, stored without specific precautions, can lose antigenicity starting from as soon as 3 months for some immunoreactions. This seems to occur especially for nuclear and membrane antigens which mostly require the use of heat-based pretreatment for antigen retrieval (Grillo et al. 2015). The analysis of different methods of preservation/storage of precut sections for an extended period of time (up to 3 years) has shown that only storage at 4° Celsius is able to effectively counteract this phenomenon while other storage systems such as immersion in liquid paraffin of precut sections or storage in cardboard/plastic box sheltered from light and air, do not appear to be as effective.

Considering this, the paraffin block appears to be the best tool to preserve tissue antigenicity. Indeed, immunohistochemical tests performed on formalin fixed and paraffin embedded (FFPE) tissue stored for several decades have shown good preservation of most tissue antigens that are frequently evaluated by immunohistochemistry. Exceptions include a few antigens, such as Ki67, CD31, CD34 and CD45, with nuclear or cytoplasmic location, which routinely require heat-based antigen retrieval (Grillo et al. 2017a). In particular, reduction of antigenicity for Ki67, a nuclear protein expressed in cells which enter the cell cycle, is detected on FFPE tissue samples after 10–15 years of storage. This length of time is important when considering that scientific research, especially in rare diseases, is carried out on archive material stored even for several decades making FFPE block collections an irreplaceable resource for retrospective research studies (Gnanapragasam 2010; Fairley et al. 2012).

In some fields, this may also be relevant in clinical practice. An example is the evaluation of the proliferation index by Ki67, central to the classification of neuroendocrine neoplasms (NEN) and fundamental in prognostic assessment and therapeutic management (Barbieri et al. 2014). This proliferation-based grading system was introduced relatively recently and, as well-differentiated NENs are neoplasms which may follow an indolent course with possible long survival, some patients may not have benefitted from correct classification in the past. Furthermore, patients may show metachronous relapses at different sites, even many years after diagnosis and comparison of the proliferative index (grade) on archival material, stored for several years (and not previously assessed), may be required (Grillo et al. 2016a, 2017b). In these cases, while having shown that the revaluation of archive material is feasible and effective (Grillo et al 2016b), it is necessary to ensure that the evaluation of Ki67 is reliable and not altered by tissue ageing.

On the basis of previous experiences with restoration of antigenicity in FFPE tissue (Grillo et al. 2017a), our aim is to comprehensively test and identify methods which may be used to obtain Ki-67 immunohistochemical reactions of good quality from old archival FFPE blocks.

## Materials and methods

### Case collection

All procedures and studies involving human participants were in accordance with the ethical standards of the institutional research committee and with the 1964 Helsinki Declaration and its later amendments. All samples were anonymous and no medical or survival data were collected.

### Re-embedding of samples

**Fig. 1:**
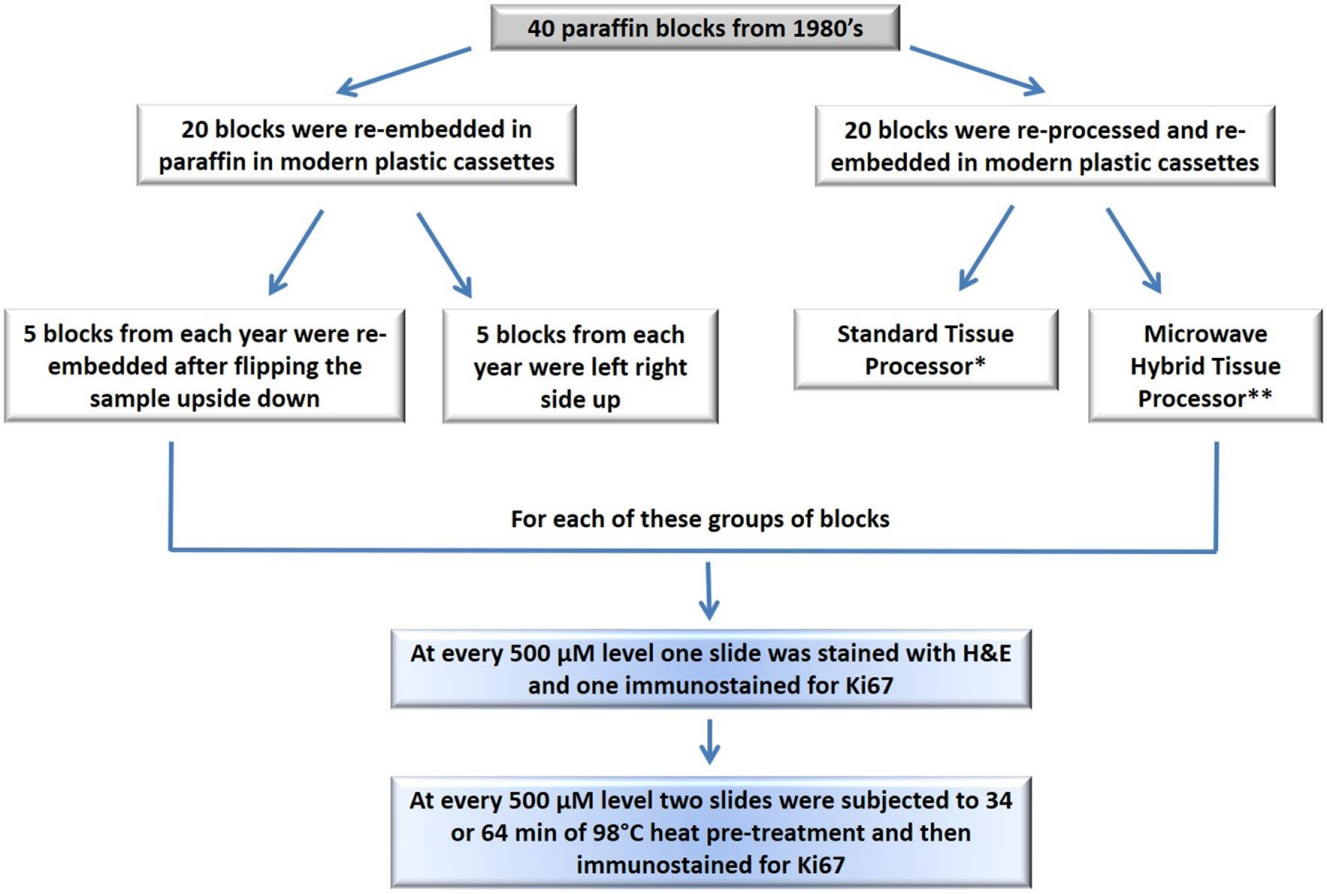
Flow chart detailing the design of the study. H&E: Haematoxylin and Eosin, *standard processing using Leica Biosystems ASP6025S (Leica Microsystems srl, Wetzlar, Germany); **Milestone LOGOS microwave hybrid tissue processor (Milestone Medical, Bergamo, Italy)

From the archives of the Anatomic Pathology laboratory of the Policlinico San Martino Hospital and University of Genoa, Italy, 20 paraffin blocks from routine histology cases from 40 years ago (1980′s) were selected (see Fig. 1 for complete protocol scheme). All FFPE samples were stored in closed cardboard boxes, protected from light, in a dry dedicated deposit at constant room temperature between 20 and 25 °C. The samples were chosen so that the paraffin blocks were of a minimum thickness of 2.5 mm.

The old paraffin blocks had been mounted on plastic cassettes that were no longer suitable for current microtomes, therefore, samples were re-embedded in paraffin wax on new plastic mounts, and these were considered as baseline samples. Half of the samples were re-embedded by flipping the sample upside down so that the underside became the sectioning surface.

### Reprocessing of samples

A further 20 paraffin blocks from the same decade as above (1980′s) were selected (see Fig. 1 for complete protocol scheme) from the same pathology archives. The FFPE blocks were then re-processed (after de-paraffinization and re-hydration) using two different automated tissue processors: Leica Biosystems ASP6025S (routinely used in our laboratory) (Leica Microsystems srl, Wetzlar, Germany) and the Milestone LOGOS microwave hybrid tissue processor (Milestone Medical, Bergamo, Italy), a new generation processor which uses microwaves and heat during all the phases of processing. Once samples were reprocessed, they were paraffin embedded and sent to the next phase of the protocol (see following section).

### Methods to Increase Ki67 Immunointensity

From each paraffin block, sections were cut, one of which was stained with hematoxylin and eosin (H&E) and one immunostained for Ki67 (clone 30.9) (Ventana Medical Systems, Roche Diagnostics Division, Hoffman La Roche Ltd, Basel, Switzerland). Sections for immunohistochemistry were prepared following previously published recommendations (Gambella et al. 2017). Following this, further sections were cut for Ki-67 staining at levels of 500 µM and 1000 µM into the block, in order to verify whether, in the deepest part of the paraffin block, antigenicity of the tissue was better preserved compared to the more superficial portions.

Every time sections were cut (at initial baseline analysis, at 500- µM depth and at 1000- µM depth), 2 sections per level were immunostained with Ki-67 using different heat-based antigen retrieval protocols: (a) standard 34 min at 98 °C; (b) prolonged 64 min at 98 °C which is not routinely used for Ki67 antigen retrieval.

We decided to limit analysis at a depth of 1000 µM without cutting the deepest portion of the FFPE blocks because, by 1000 µM, the central part of the block had already been reached both in blocks which had been re-embedded right side up and flipped upside down.

An integrated on-slide control for Ki-67 reaction, obtained from recently prepared FFPE tissue with standardized preanalytical factors, was used as reference and comparison for positive immunostaining (Bragoni et al. 2017). All immunohistochemistry was performed using the BenchMark Ultra (Ventana Medical Systems, Roche Diagnostics Division, Hoffman La Roche Ltd, Basel, Switzerland) automated immunostainer.

**Fig. 2:**
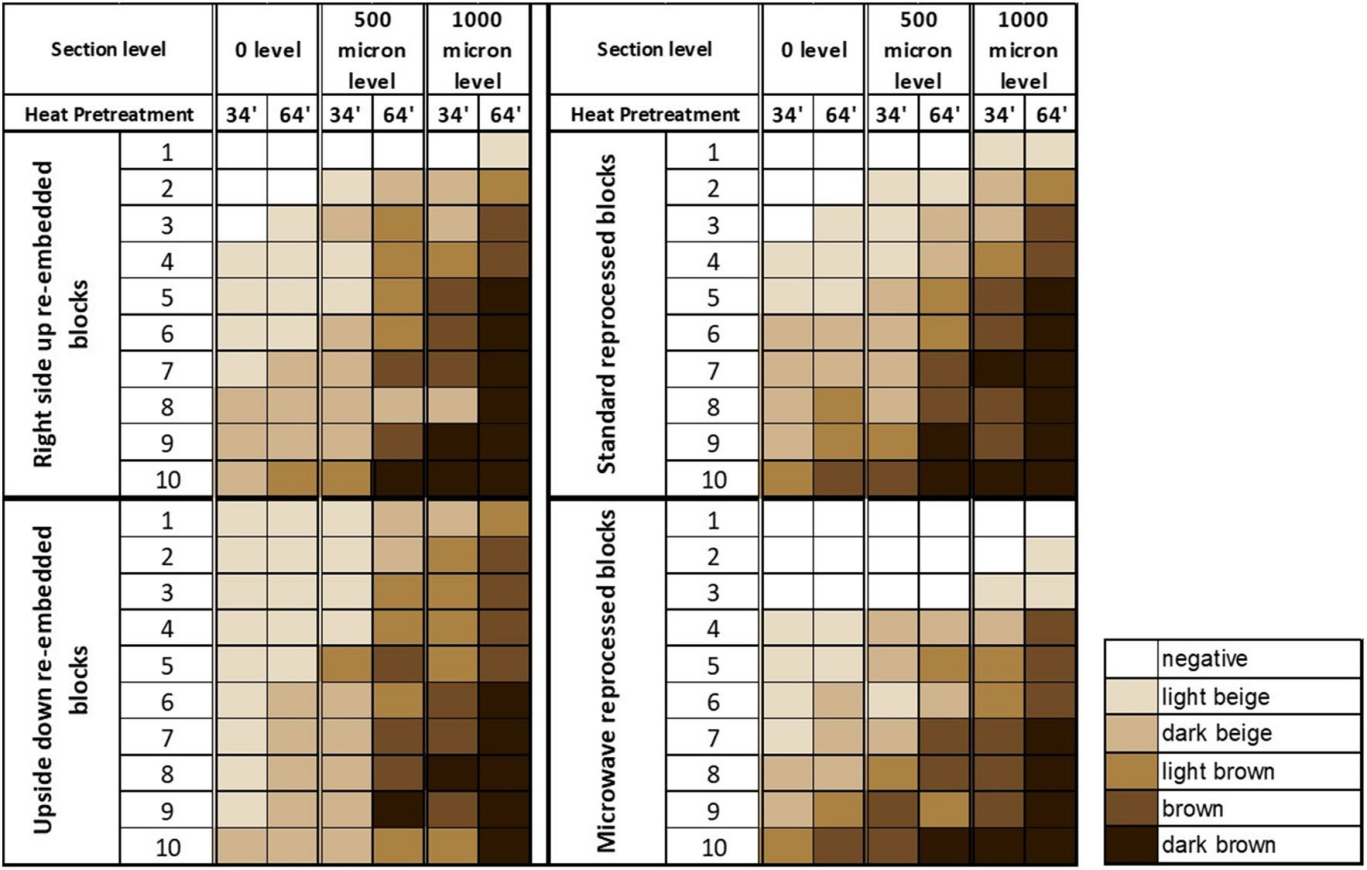
Case evaluations according to colour coded reference system

Evaluation of immunoreactions was performed, on portions of tissue with proliferating areas, either normal or neoplastic. Immunoreaction intensity was graded as negative (no nuclear positivity in areas which should show proliferation) or positive with various gradients spanning from light beige to dark brown (Adsay 2012). A colour-coded reference system was adopted as shown in Fig. 2. For each case the maximum staining intensity per slide was reported and comparisons made with the baseline Ki67 section and the positive on-slide control.

All microscopy images were acquired using an Olympus U-MDO B3 optical microscope, objective lens 2 × magnification plan 0.10 and 40 × magnification plan 0.6, with digital camera Olympus DP70 (12.5 million-pixel) and acquisition software DPController 2004 (Olympus Corporation, Tokyo, Japan).

## Results

### Evaluations on re-embedded paraffin blocks

(a) Comparison of Ki67 expression between on-slide control and initial section at standard heat pretreatment

**Fig. 3:**
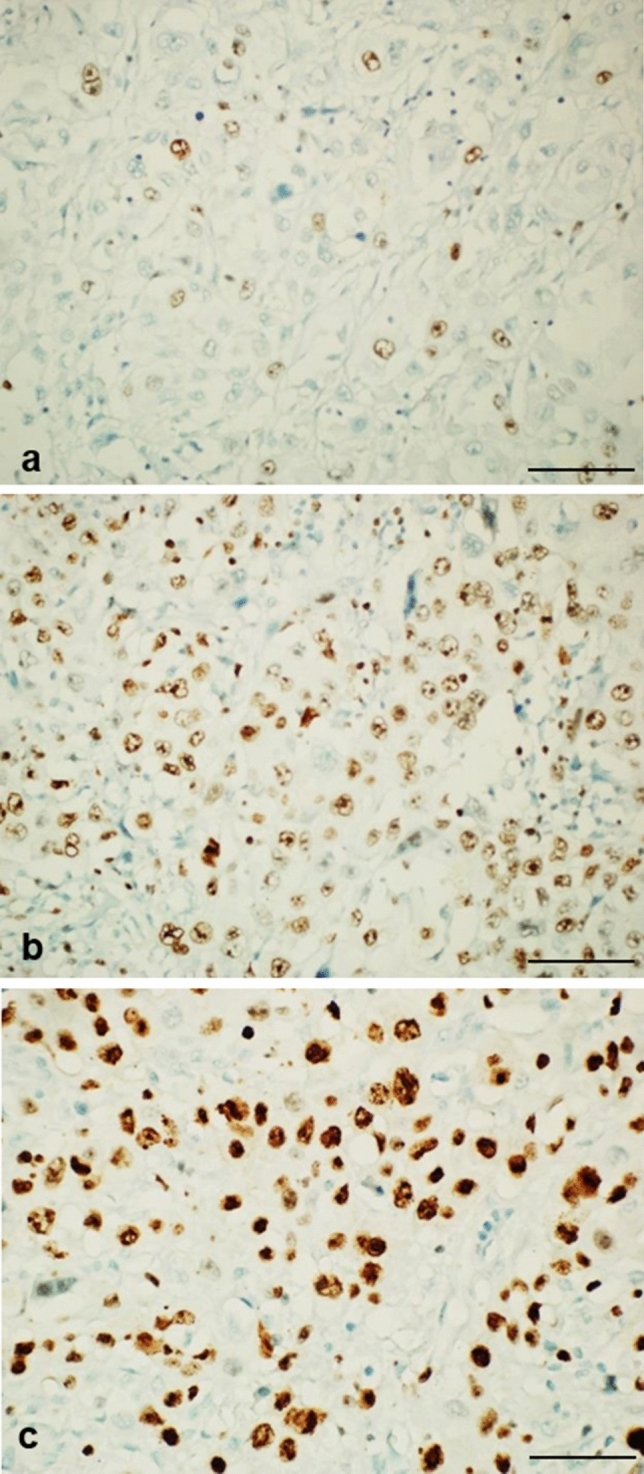
Ki67 immunostained sections from the same case with no flipping or re-processing of block. **a** Initial KI67 stained section with standard 34-min heat pretreatment showing faint, from unstained, light beige to dark beige nuclei. **b** Ki67 stained immunoreaction after deeper (500 µM) sectioning and prolonged 64-min heat pretreatment showing dark beige nuclei. **c** Ki67 stained immunoreaction after deeper (1000 µM) sectioning and prolonged 64-min heat pretreatment showing dark brown nuclei. Scale bar 50 µm

All sections from all cases showed an evident reduction of immunostaning intensity ranging from completely negative to a maximum of light or dark beige staining colour (Figs. 2 and 3a) at initial section. No intensely staining, brown nuclei, were observed compared to the on-slide reference control.

(b) Comparison of Ki67 expression between right side up and upside down re-embedded blocks at standard heat pretreatment

No significant differences between right side up and upside-down paraffin embedded blocks were noted in Ki67 immunostaining intensity. The initial sections showed the same light coloured, beige staining as above (Fig. 2).

(c) Comparison of Ki67 expression between initial section, 500-µM level and 1000-µM level at standard heat pretreatment

Ki67 immunostaining showed improved intensity when deeper levels were cut (Fig. 2) at standard 34-min heat-based pretreatment. A slight improvement in staining intensity, with two cases out of 20 (10%) in which nuclei were light brown coloured, was observed when a first level of 500-µM waste was reached. Staining intensity showed further improvement in the deepest part of the paraffin block, at the 1000-µM level, with 9 cases out of 20 (45%) reaching a brown/dark brown staining intensity.

(d) Comparison of Ki67 expression between standard 34 min and increased 64-min heat-based antigen retrieval

The prolonged heat-based treatment protocol (64 min) improved staining intensity at each level of sectioning with best results obtained at 1000-µM level: 17/20 (85%) cases showed brown and dark brown nuclear expression (Figs. 2 and 3b, c). Normal, nonproliferating tissues (e.g. nonbasal layers of squamous epithelium), did not show any artefactual Ki67 positive nuclei.

### Evaluations on reprocessed paraffin blocks

Comparison of Ki67 expression between re-embedded and re-processed sections (standard and microwave assisted) at standard heat pretreatment and 64-miute pretreatment at different levels.

The re-processed cases did not show significant improvement in Ki67 immunointensity as compared to just re-embedding (Fig. 2) at initial sectioning and with standard pretreatment. When, deeper sections were cut and longer heat pretreatment times were used, a minimal increase in immunoreactivity was observed, which however did not substantially differ from the nonreprocessed cases. In detail, 4 out of 10 cases showed a brown/dark brown staining intensity starting from the 500-µM level (compared to 7 out of 20 nonreprocessed cases—right side up or flipped upside down—and 3 out of 10 of the microwave-assisted reprocessing).

On the other hand, at the 1000-µM level, effects of re-processing on immunostaining were negligible (brown/dark brown staining intensity in 17 out of 20 nonreprocessed cases—right side up or flipped upside down—and 15 out of 20 of the standard and microwave-assisted reprocessing).

### Sample heterogeneity and preservation

**Fig. 4:**
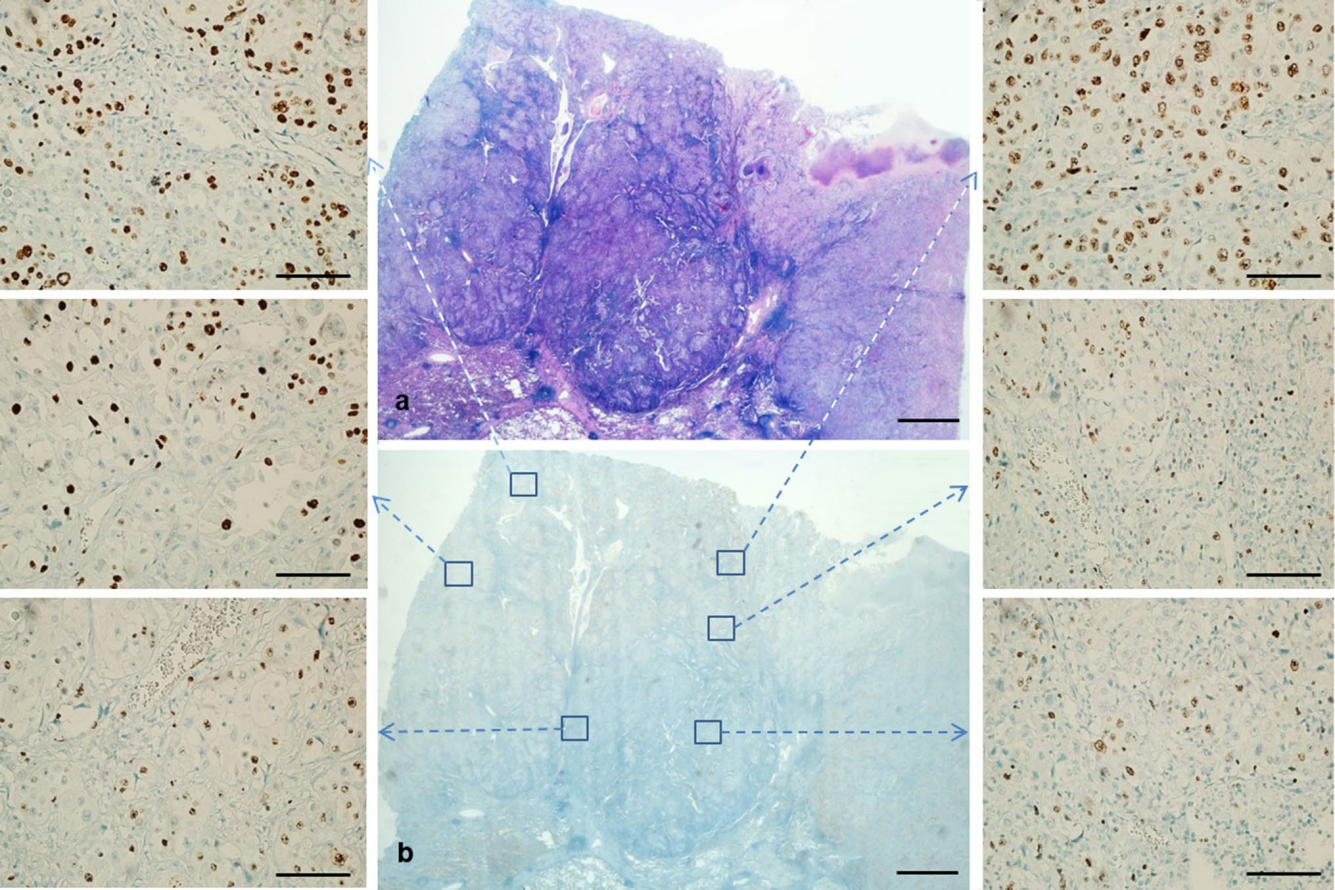
Ki67 immunoreaction heterogeneity, showing differences in Ki67 immunointensity between periphery and central portions (see boxes). **a** Haematoxylin and eosin; **b**: Ki67 (scale bar 2500 µm). Ki67 in boxes (scale bar 50 µm)

In all large samples (Fig. 4), Ki67 staining intensity showed heterogeneity between the periphery of the sample and the central area. Indeed, the periphery showed a rim of more intensely coloured nuclei as compared to the center, and this was seen in all samples, independent of performed procedures.

No significant problems in detachment were observed in all our cases, even in those which were intensively treated.

## Discussion

Pathology archives are a treasure trove of paraffin embedded tissue spanning many years and covering a wide variety of tissues and diseases. However, the use of such material requires archives of stable, well-preserved samples. Although the preanalytical phase is now under the spotlight and is considered a crucial step in maintenance of tissue antigenicity, as well as RNA and DNA preservation, this is not true for old archival samples. Furthermore, the effect of ageing on FFPE tissue samples is poorly studied (Dwork et al. 1998; Littlekalsoy et al. 2007). A previous study (Grillo et al. 2017a) (within our quality assurance initiative), performed on old archival blocks, focused on antigen loss of expression in FFPE tissue, stored for several decades and spanning a panel of frequently used immunomarkers. Having shown that antigen loss may ensue over time, we decided to focus on testing different procedures that can help in antigen unmasking of Ki67 which, in our experience, was one of the most severely affected antigens.

Our comprehensive analysis has demonstrated that:

1. Tissue samples fixed in formalin and embedded in paraffin for a period of 40 years show a significant reduction in antigenicity for Ki67.
2. As already shown in a previous study (Grillo et al. 2017a) and confirmed in this present contribution, the use of prolonged heat-based antigen retrieval, is a valid tool for restoring antigenicity of Ki67. Heat-based antigen retrieval is effective in breaking nonspecific covalent bonds between tissue antigens and formalin, thereby unmasking these antigens for antigen–antibody binding in immunohistochemistry. As time passes, oxidation contributes to antigen masking (Blind et al. 2008) and more lengthy times of heat-based antigen retrieval become necessary.
3. The flipping upside down of FFPE samples does not effectively contribute to Ki67 antigen retrieval. This is probably due to the fact that the factors which cause antigen loss/masking over time (e.g. oxidation) have an effect on both sides of the paraffin block.
4. The sectioning of samples at 500 and 1000-µM levels is effective in most cases. This may be explained considering that the deepest parts of the FFPE blocks are better protected from the aforementioned factors (mainly oxidation by contact with air) which affect antigenicity.
5. The combination of deeper sectioning of samples, together with prolonged heat-based retrieval, shows the best results with regards to increasing Ki67-staining intensity. This protocol does not artefactually increase Ki67 as nonproliferating normal cells were still Ki67 negative. Of note is that the deep sectioning of FFPE blocks leads to waste of tissue and therefore can be carried out only on surgical tissue samples with a thickness of at least 2 mm.
6. Reprocessing, either by standard or modern microwave assisted processors, does not comprehensively affect Ki67 restoration. The basis of this trial was that antigen retrieval for Ki67 is heat based and, as microwave processing uses heat to speed processing, this may have a double effect on antigen retrieval. In this context, standard reprocessing was used to verify whether any form of re-processing could break oxidative bonds and unmask antigens. Neither of these situations proved to be significantly more effective than the combination of deeper sectioning of samples with prolonged heat-based retrieval. A possible explanation is that tissue becomes excessively treated, as it is de-paraffinized and rehydrated to formalin and subsequently reprocessed, thus leading to further formalin crosslinking. Another possibility is that during re-processing (standard and microwave) the maximum temperature which is reached is 65 °C, which is probably insufficient for antigen restoration considering that heat-based antigen reaches 98 °C.
7. In large samples a problem of immunoreaction zonality remains, even in samples subjected to a cutting depth of 1000 µM and with prolonged thermal unmasking. The periphery of the section shows more intense positivity compared with the central part. This is often seen in routine diagnostics for various antibodies (e.g. mismatch repair proteins) and two hypotheses have been proposed to explain this phenomenon: (i) an antibody concentration gradient with a more concentrated outer rim compared to the central part which is unlikely in this scenario as the automated immunostainers are designed to counteract this; (ii) variable fixation in the periphery compared to the central area and this is probable as the preanalytic phase, which we know is a crucial step, was not as controlled as it is today.

A limit of this study is that the whole study has focused on Ki67 restoration but these methods may not readily apply to other antigens. Indeed, specific protocols may be required for other problematic antigens. A further limit, but also a possible explanation of variable results between different FFPE blocks, is that we have no information on the differences in preanalytical factors between samples and this may have influenced some evaluations. An intrinsic limitation in the use of a deep-sectioning protocol is the impossibility of its use in small biopsy samples or tissue microarrays, for which deep sectioning may lead to complete tissue loss. Lastly, the study has focused on using automated immunostainers (which are now widely used in clinical and research labs) while different heat-based antigen retrieval systems, such as autoclave, microwave, pressure cooker and laboratory thermostatic baths, can also be used. With this in mind, the basic principle that an increase in heat for antigen retrieval to give better results in immunostaining, requires adaptation to other heat-based retrieval systems.

In conclusion this study, following our quality assurance program in our pathology laboratory, suggests a methodology based on both deep sectioning and prolonged heat pretreatment, for restoration of Ki67 antigenicity in old archival tissue samples either for diagnostic (re-evaluation) or retrospective research studies.

## Data Availability

All data is available upon request.
